# An International Marine-Atmospheric ^222^Rn Measurement Intercomparison in Bermuda Part II: Results for the Participating Laboratories

**DOI:** 10.6028/jres.101.006

**Published:** 1996

**Authors:** R. Collé, M. P. Unterweger, J. M. R. Hutchinson, S. Whittlestone, Georges Polian, Bénédicte Ardouin, Jack G. Kay, James P. Friend, Byron W. Blomquist, Wolfgang Nadler, Thomas T. Dang, R. J. Larsen, A. R. Hutter

**Affiliations:** National Institute of Standards and Technology, Gaithersburg, MD 20899-0001, USA; Australian Nuclear Science and Technology Organization, PB1, Menai, NSW 2234 Australia; Centre des Faibles Radioactivités, Laboratoire Mixte, C.N.R.S-C.E.A., 91198 Gif-sur-Yvette, France; Atmospheric Chemistry Laboratories, Department of Chemistry, Drexel University, 32nd & Chestnut Streets, Philadelphia, PA 19104, USA; Environmental Measurements Laboratory, U.S. Department of Energy, New York, NY 10014, USA

**Keywords:** air, environment, intercomparison, marine atmosphere, measurement, radon-222

## Abstract

As part of an international measurement intercomparison of instruments used to measure atmospheric ^222^Rn, four participating laboratories made nearly simultaneous measurements of ^222^Rn activity concentration in commonly sampled, ambient air over approximately a 2 week period, and three of these four laboratories participated in the measurement comparison of 14 introduced samples with known, but undisclosed (“blind”) ^222^Rn activity concentration. The exercise was conducted in Bermuda in October 1991. The ^222^Rn activity concentrations in ambient Bermudian air over the course of the intercomparison ranged from a few hundredths of a Bq · m^−3^ to about 2 Bq · m^−3^, while the standardized sample additions covered a range from approximately 2.5 Bq · m^−3^ to 35 Bq · m^−3^. The overall uncertainty in the latter concentrations was in the general range of 10 %, approximating a 3 standard deviation uncertainty interval. The results of the intercomparison indicated that two of the laboratories were within very good agreement with the standard additions, and almost within expected statistical variations. These same two laboratories, however, at lower ambient concentrations, exhibited a systematic difference with an averaged offset of roughly 0.3 Bq · m^−3^. The third laboratory participating in the measurement of standardized sample additions was systematically low by about 65 % to 70 %, with respect to the standard addition which was also confirmed in their ambient air concentration measurements. The fourth laboratory, participating in only the ambient measurement part of the intercomparison, was also systematically low by at least 40 % with respect to the first two laboratories.

## 1. Introduction

An international measurement intercomparison of instruments used to measure ^222^Rn in marine atmospheres was organized by Drexel University and conducted in Bermuda in October 1991. This paper, the second of two in a series, provides the intercomparison results for the participating laboratories. The intercomparison exercise consisted of two components: (1) measurement comparisons among four laboratories of commonly sampled ambient air over approximately a 2-week test period; and (2) measurement comparisons between three of these four laboratories of a select number of introduced samples with known, but undisclosed (i.e., “blind”) ^222^Rn activity concentration that could be related to U.S. national ^226^Ra standards.

The first paper in this series [1] provides detailed descriptions of the experimental arrangements, the ^226^Ra source calibrations used to obtain known ^222^Rn activity concentrations, the methodology for the standardized sample additions, and the protocol for the experimental aspects of the intercomparison.

The standardized sample additions were provided by the National Institute of Standards and Technology, and were made with a commercially-available, ^226^Ra source that was calibrated by NIST in terms of the available ^222^Rn concentration as a function of constant flow rate through the source. The source was employed in conjunction with a specially-designed manifold, also provided by NIST, that allowed the determination of well-known dilution factors for the standardized sample additions which were introduced into a common streamline on a sampling tower used by the participants for their measurements. Several confirmatory measurements were also performed during the course of the intercomparison by collecting “grab samples” from the manifold and returning them to NIST for assaying the ^222^Rn activity concentrations. These confirmatory measurements were made to insure that the radium source and manifold were not performing differently at the test site in Bermuda as at NIST where the calibrations were made.

The participating laboratories in the intercomparison were:

Centre des Faibles Radioactivités, Laboratoire Mixte, C.N.R.S-C.E.A., Gif-sur-Yvette, France

Environmental Measurements Laboratory, U.S. Department of Energy, New York, NY

Australian Nuclear Science and Technology Organization, Menai, NSW, Australia

and

Drexel University, Department of Chemistry, Philadelphia, PA

(hereafter referred to as Lab F, Lab E, Lab A, and Lab D, respectively). The latter three laboratories (E, A, and D) performed simultaneous measurements from a common stream line on an ambient air sampling tower. Lab F, in contrast, sampled ambient air nearly adjacent to the inlet of the sampling tower and, therefore, did not participate in the intercomparison of standardized sample additions, but only in the intercomparison of ambient air measurements.

**Fig. 1:**
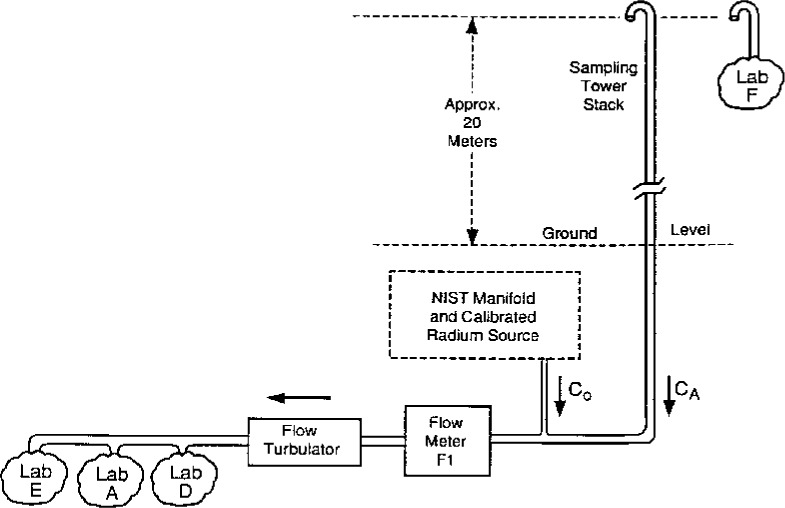
Schematic diagram of the experimental configuration used for the measurement intercomparison showing the relative sampling locations for the participating laboratories.

The experimental configuration used for the intercomparison is illustrated in Fig. 1. Additional details may be found in Collé, et al. [1]. The mean ^222^Rn activity concentrations (over some given sampling-time interval) used for the intercomparisons, and as labelled in the figure, are defined [1] as follows:

*C*_0_: is the mean concentration provided by NIST for the standardized sample additions (independently evaluated for each of their measurement/sampling intervals);
*C*_A_: is the mean concentration in ambient air; and
*C*_1_: is the mean concentration in the main stream line sampled by Labs E, A, and D (e.g., *C*_1_ = *C*_0_ + *C*_A_).

## 2. Measurement Methods of the Participating Laboratories

Each of the instruments and measurement methodologies employed by the four participating laboratories were based on different analytical approaches. The main characteristics for each are summarized in Table 1. The tabulation is comprised of data and information supplied by the participating laboratories. Such a comparative summary is, of course, limiting inasmuch as it is nearly impossible to fully and adequately describe and characterize such complex instruments and methods by constraining them to the broad, general categories of characteristics given in Table 1. Readers interested in further detail on any of the instruments or methods are therefore encouraged to consult the original references or to obtain additional information directly from the respective participating laboratory.

The instrumentation used by Lab E was based on a two-filter ^222^Rn measurement method [2]. Simply, the method consists of the following. Sample air is pumped through two filters in series that are separated by a large decay volume to permit decay of the radon. The first filter removes all progeny in the ^222^Rn subseries (^218^Po-^214^Pb-^214^Bi-^214^Po). In the decay volume, some of the ^222^Rn in the flow stream undergo radioactive decay, producing new progeny. These progeny are collected on the second, downstream filter and measured with an α-sensitive scintillation detector. The ^222^Rn concentration in the sample air is derived from the resulting α-activity counting rate. The Lab E instrument consists of a 500 L cylindrical decay chamber and utilizes flow rates typically ranging from 350 L · min^−1^ to 400 L · min^−1^. The instrument is fully automated. The second filter is configured as a ribbon, capable of forward and backward motions that allow rewinding of the filter [3]. For this intercomparison exercise, the instrument used sampling intervals of 1 h to collect the ^222^Rn progeny after which the filter was transported to a ZnS(Ag) detector for measurement intervals of 1 h. While one section of the filter is being counted, another section is used in sampling. Additional characteristics and performance data are summarized in Table 1.

The instrument used by Lab A was modelled on that described by Whittlestone [7]. The methodology is based on a modification of the two-filter method that incorporates an aerosol particle generator. Its main principles of operation are as follows. Sample air was continuously pumped at a flow rate of 400 L · min^−1^ through a drum of volume 200 L to allow decay of short-lived (55 s half life) ^220^Rn (thoron), then through an absolute filter into a large plastic chamber of volume 2000 L. Sub-micron particulate condensation nuclei (CN) were injected into the chamber so that the ^222^Rn progeny become attached to the CN rather than to the chamber walls. The attached progeny, after an average residence time of 50 min in the chamber, were filtered onto a membrane filter that was continuously monitored with a ZnS(Ag) scintillator. Average ^222^Rn concentrations were inferred from 30 min accumulations of counts from the decay of the ^222^Rn progeny on the filter. A calibrated CN counter was incorporated into the system to account for changes in the detector efficiency as a function of CN concentrations. Table 1 contains additional information on the instrument’s performance and characteristics.

The ^222^Rn measurement results of Lab F were based on inferring ^222^Rn concentrations from collection and assay of the short-lived ^222^Rn progeny that are attached to aerosols in ambient air. The methodology assumes that the ^222^Rn and its progeny are in radioactive equilibrium (or are in a state of known equilibrium ratio). The assumption of radioactive equilibrium (with an equilibrium ratio of unity) is usually considered to be valid at sampling locations distantly far from continental sources of radon and not influenced by local land masses. The instrument used by Lab F consisted of a large circular filter fixed on a rotating disk that divided the filter into 12 sampling locations. The instrument was automated for simultaneous sampling and measurement intervals of 2 h. After an aerosol sample was collected at one sampling location on the filter, it was automatically rotated to an α-sensitive scintillation detector for counting while the next sample was collected. As for previously described instruments, performance data and additional details on this instrument may be found in Table 1.

The methodology utilized by Lab D was a non-filter method based on separating radon from air samples and subsequently assaying it in one of six Lucas-type ZnS(Ag) scintillation cells. The instrument was fully automated and operated with the following sequential steps: An air sample at a flow rate of 28 L · min^−1^ was aspirated into the instrument, compressed and dried, and flowed through a cooled charcoal trap where the radon was separated from the air stream by adsorption. Following a sampling interval of 2 h, the collected radon was heat- and vacuum-transferred to a secondary cooled charcoal trap to effect separation with other gases contained in the main charcoal trap. A pre-evacuated scintillation cell, having a pre-determined background counting rate, was then filled by transferring the radon from the secondary trap. Sample separation and processing times were of the order of 2.5 h and, with the sampling interval of 2 h, resulted in a sampling frequency of about one sample every 4.5 h. Reported ^222^Rn activity concentrations were derived from the total α-counting rates from the scintillation cells after about 4 h when the ^218^Po and ^214^Po progeny follow ^222^Rn decay in transient equilibrium. Again, further detail and performance data are provided in Table 1.

## 3. Reported Measurement Results

**Fig. 2:**
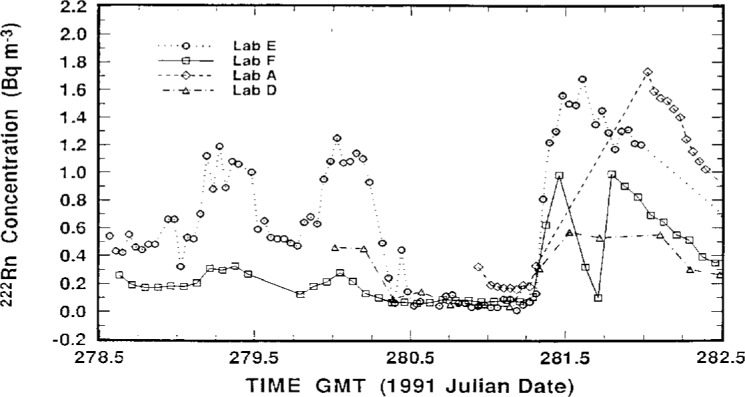
Mean ^222^Rn concentrations (*C*_A_) for ambient Bermudian air reported by the four participating laboratories over the course of 4 days prior to the standardized additions.

**Fig. 3:**
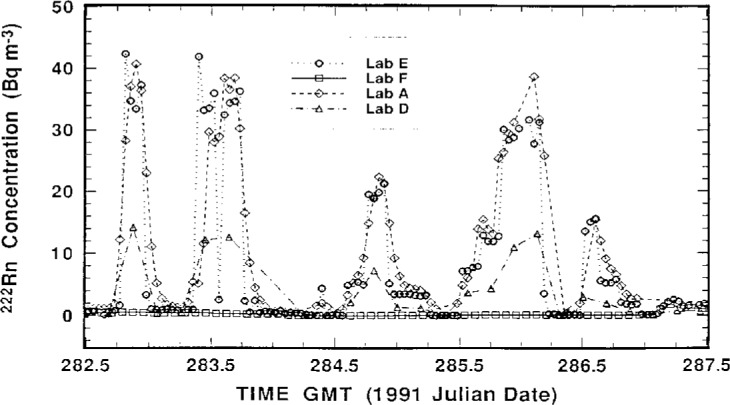
Mean ^222^Rn concentrations (*C*_0_ + *C*_A_) reported by the four participating laboratories during the course of the standardized additions.

**Fig. 4:**
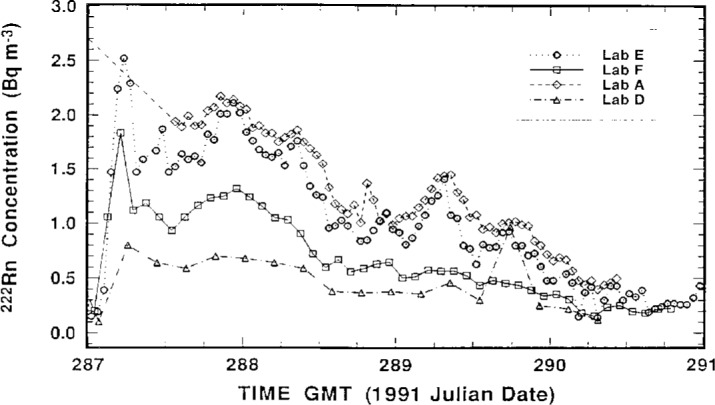
Mean ^222^Rn concentrations (*C*_A_) for ambient Bermudian air reported by the four participating laboratories over the course of 4 days following the standardized additions.

The measurement results of the participating laboratories, as reported by them, over the intercomparison period October 5–17, 1991 are summarized in Figs. 2 through 4. The figures provide reported values of the mean ^222^Rn activity concentrations over their individual sampling/measurement intervals for both the standardized sample additions (*C*_1_) and for the ambient air measurements (*C*_A_). The times, Greenwich Mean Time (GMT) in units of 1991 Julian date, correspond to approximate mid-point times for each sampling/measurement interval. The concentrations reported by each laboratory were converted to common units of Bq · m^−3^ for comparison. Figure 2 provides the first four days of ambient air measurements; Fig. 3 gives the reported concentrations over the course of the 15 standard additions; and Fig. 4 shows the results of four days of ambient measurements following the standard addition period.

A complete tabulation of the reported values for all four laboratories is provided in Table A of Appendix A to this paper.

As indicated previously, the results for Lab F represent only measurements of *C*_A_. Lab F reported both mean activity concentrations for the measured 212Pb daughter activity and the inferred ^222^Rn activity for intervals of 2 h. Only the reported ^222^Rn concentration values are reported and treated here. Lab F separately noted two conditionals: ^222^Rn concentration values that corresponded to ^212^Pb concentrations having what were considered by Lab F to be abnormally high values due to local land influences; and values suspected to have been influenced by rainfall (see Appendix). These conditions affected only a small fraction of the data values: 13 and 5, respectively, out of a total of 142 reported values. No effort was made to separately treat these conditional values. The uncertainties associated with the ^222^Rn concentrations in the range of about 0.07 Bq · m^−3^ to 0.2 Bq · m^−3^ was estimated and reported to be approximately plus or minus 20 %. This uncertainty was reported to correspond to two standard deviations for an assumed Poisson-distribution statistical “counting error” (based on the square root of the total number of detected counts) as well as contributions due to the uncertainties in detection efficiency and flow-rate measurements.

**Fig. 5:**
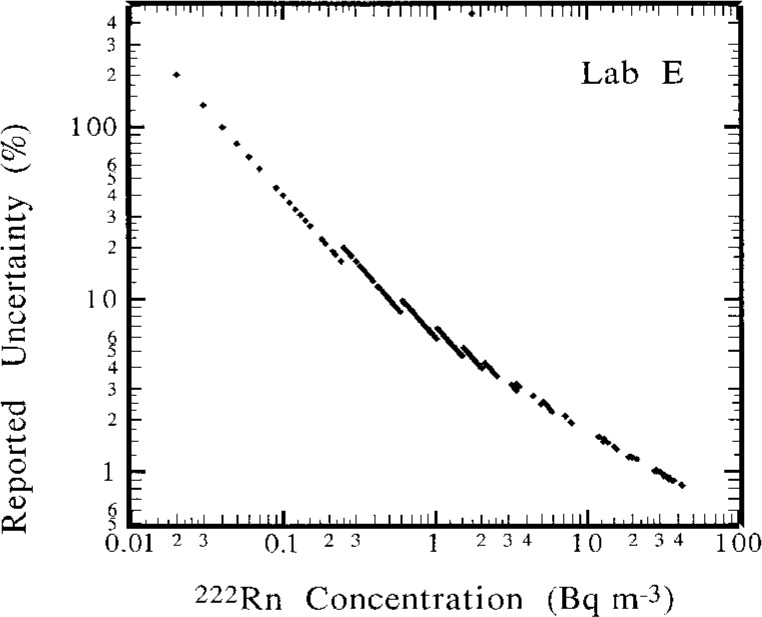
Lab E reported uncertainties as a function of the ^222^Rn concentration. The uncertainty, expressed in percent, corresponds to just a 1 standard deviation interval for an assumed Poisson-distributed statistical “counting error.”

The results in Figs. 2 through 4 reported by Lab E are for sampling/measurement intervals of 1 h. The uncertainties for these values, which were reported to correspond to a one standard deviation interval for the assumed Poisson-distributed statistical “counting error,” are shown in Fig. 5 where the reported uncertainty, expressed in percent, is given as a function of the mean activity concentration. The data plotted in Fig. 5 are reported values for all of the results reported for Lab E. The functional form of the shown data is, of course, just directly proportional to *C*^−1/2^ where *C* is the reported concentration (since the variance equals the mean *C* with the assumption of a Poisson distribution). The minor discontinuities in the plotted data arise from the limited number of reported significant figures in the original data set. As indicated, the reported uncertainties for just the statistical “counting error” range from well over 100 % at concentrations of a few hundredths of 1 Bq · m^−3^ to less than 1 % at concentrations of about 30 Bq · m^−3^. These uncertainties do not necessarily represent the inherent or minimum obtainable precision of the two-filter technique. Those reported here are significantly influenced by the count rate arising from a thoron (^220^Rn) contamination that is due to trace quantities of thorium in the materials used to construct their decay chamber. This thoron background is treated as part of the overall counting system background. The magnitude of this contribution may be appreciated by considering that the 39 % relative uncertainty at a concentration of 0.1 Bq · m^−3^ would decrease to 19 % in the absence of the thoron. This contamination problem can be eliminated, such as is done by Lab A, by using a plastic or fiberglass decay chamber with a conducting inner surface. It has been reported that Lab E has subsequently eliminated the thoron contamination problem by coating the welds in the decay chamber with a white epoxy [6].

**Fig. 6:**
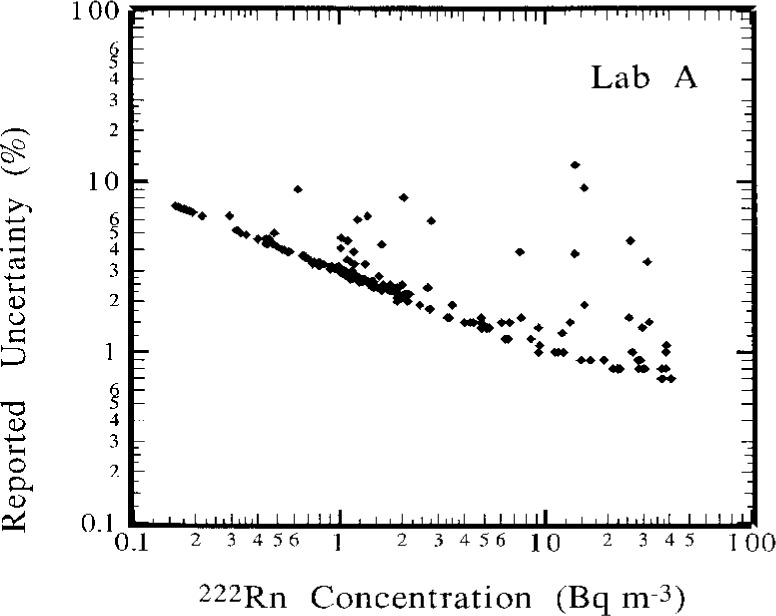
Lab A reported uncertainties as a function of the ^222^Rn activity concentration. The uncertainty, expressed in percent, corresponds to a 1 standard deviation interval for an assumed Poisson-distributed statistical “counting error” combined with a particulate concentration/detection efficiency uncertainty component (refer to text).

The results in Figs. 2 through 4 for Lab A are also for intervals of 1 h. As indicated previously, however, their instrument recorded continuously and gave averaged results that were “smoothed” by a time constant of approximately 90 min. As a result of the smoothing, the evaluation of their results could not be as direct, or as subjectively unequivocal, as with that of other laboratories. The measurement uncertainties reported by Lab A for their data set is given in Fig. 6. This uncertainty was stated to correspond to a one standard deviation statistical “counting error” combined with the estimated uncertainty in correcting for detection efficiency variations that arise because of changes in particulate concentrations in their delay tank. The lower edge of the data set plotted in Fig. 6 corresponds to the identical kind of *C*^−1/2^ functional form described for Lab E in Fig. 5. The large positive deviations correspond to periods when the delay chamber condensation nuclei concentration was low because of a power supply fault which resulted in a detection efficiency well below optimum. Over the course of the intercomparison, the radon concentration ranged mainly between 0.2 Bq · m^−3^ to 40 Bq · m^−3^. The uncertainties for Lab A in this concentration range varied from 12 % to 0.7 %, which is comparable with those of Lab E (20 % to 0.7 %). However, the uncertainties at lower concentrations were markedly less for Lab A, presumably because of the thoron contamination in the Lab E decay chamber. For example, at a concentration of 0.1 Bq · m^−3^ the uncertainty for Lab A was 8 %, whereas that for Lab E it was 40 %.

The reported measurement results for Lab D given in Figs. 2 through 4 are mean concentrations averaged over sampling intervals of 2 h. Associated relative uncertainties for a one standard deviation interval were reported to range from ± 3.4 % to 3.9 % for the entire data set, and include contributions from 1) the measurement variability which was stated to have a relative magnitude of about 2.8 % across the range of observed activity concentrations; 2) from the uncertainty associated with a ^226^Ra/^222^Rn reference standard used for calibration; and 3) from the flow meter uncertainty over the range of flow rates used.

## 4. Intercomparison of Standardized Sample Additions

Derivation of the mean ^222^Rn activity concentrations *C*_0_ for the standardized sample additions provided by NIST to the participating laboratories is treated *in extenso* in Collé et al. [1]. Fifteen additions, each having a duration of 4 h (except for #13 which was 3 h), were provided over the period October 9–13, 1991. They are summarized in Table 2. One of them (addition #4) had to be discarded from the analysis because of an experimental blunder. The activity concentrations *C*_0_ were derived independently from well-known dilution factors that were in turn obtained from simultaneous flow-rate measurements for each sampling/measurement interval (from some given start time *t*_a_ to a stop time *t*_z_) for each participating laboratory. Lab E, with well-defined *t*_a_ to *t*_z_ sampling intervals of 1 h, thus received over the course of the 14 valid additions, 53 standardized samples for comparison. The Lab A additions, because of its averaged “smoothed” measurement results, utilized the mean *C*_0_ for the entire 4 h or 3 h *t*_a_ to *t*_z_ sample intervals. Lab D, which had only one 2 h *t*_a_ to *t*_z_ sampling/measurement interval enveloped within each 4 h or 3 h sample interval, therefore, also received 14 standardized samples for comparison. The concentrations *C*_0_ for the intercomparisons ranged from approximately 2.5 Bq · m^−3^ to 35 Bq · m^−3^. Based on a very detailed uncertainty analysis, the overall propagated uncertainty in *C*_0_ was in the range of 6 % to 13 % for a 3 standard deviation uncertainty interval [1].

### 4.1 Lab E Results

**Fig. 7:**
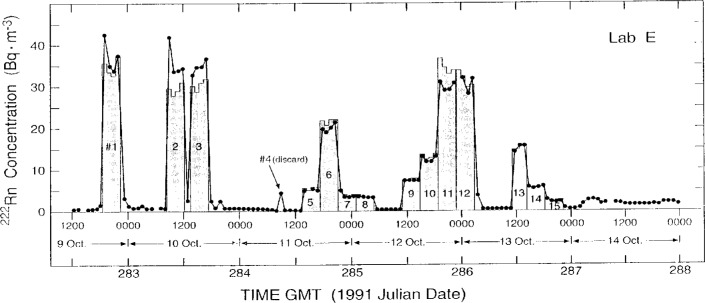
Lab E measurement results for the mean ^222^Rn activity concentration *C*_1_ (plotted data points) compared to the standardized sample concentrations *C*_0_ provided by NIST (shaded areas). The comparisons exclude contributions from the ambient concentrations *C*_A_ (refer to text).

**Fig. 8:**
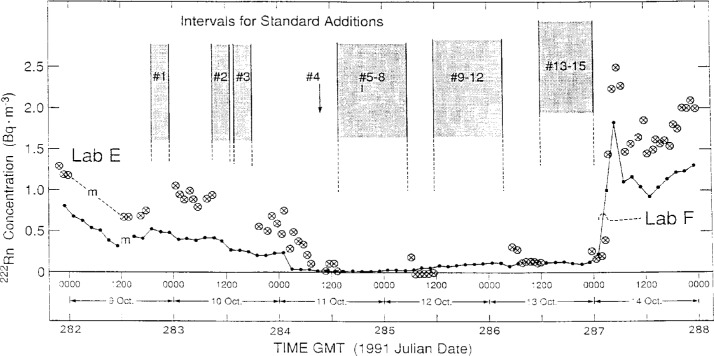
Measurement results by Lab F (solid line) and Lab E (crossed circles) for the natural Bermudian ambient air ^222^Rn activity concentrations *C*_A_ during the time of the standardized sample addition intercomparison. The time intervals for the additions are denoted. Refer to text for details and discussion.

Table 3 contains the reported measurement results by Lab E for the mean concentrations *C*_1(E)_ in the main sampling stream line, and for comparison the mean concentrations *C*_0_ provided by NIST. The derived values of *C*_0_ are given in Collé et al. [1]. The results for *C*_1(E)_ and *C*_0_ over the entire course of the standardized sample additions are illustrated in Fig. 7. Inasmuch as, by definition, *C*_1(E)_ includes the ambient ^222^Rn activity concentration whereas *C*_0_ does not, an assumed *C*_A(E)_ was selected to compare not only the concentration ratios *C*_1(E)_/*C*_0_, but also (*C*_1(E)_ − *C*_A(E)_)/*C*_0_. The ambient concentrations *C*_A(E)_ were very approximate values selected from the Lab E data set from adjacent measurement intervals that were not believed to be influenced by the standard sample additions. The large intervals between ambient concentration measurements, which might otherwise have very suspect assumed *C*_A(E)_ values, could be somewhat verified by normalizing the Lab E results to the uninfluenced Lab F results. Figure 8 shows the results of the reported measurements of *C*_A(F)_ by Lab F (solid line) over the course of the standardized sample additions, along with the reported Lab E values (crossed circles) in the intervals not influenced by the additions. As indicated in the figure, there appears to be scant cause to suspect that there were any masked irregularities in *C*_A_ during the standardized sample additions; and it seems reasonable that assumed values of *C*_A_ could be obtained from interpolations. The particular choices of assumed *C*_A(E)_ values in Table 3 may appear to be high in comparison to “eye-smoothing” interpolations with Fig. 8 (particularly for additions #1, #2, and #3). However, the choices were deliberately conservative, such that the two ratios *C*_1(E)_/*C*_0_ and (*C*_1(E)_ − *C*_A(E)_)/*C*_0_ in Table 3 were almost extreme limits on the influence of assumed *C*_A_ values. The first ambient measurement result *C*_A(E)_ by Lab E following a standardized sample addition was invariably elevated above subsequent *C*_A(E)_ measurements. This effect is due to the continuous dilution in the decay chamber from one sampling cycle to the next. This incomplete removal of previously introduced activity is most pronounced in changes from very high standard addition concentrations to ambient levels. Based on the decay chamber volume (500 L) and flow rate (400 L · min^−1^), the removal is virtually complete within a few minutes. Conservatively, therefore, in the absence of any other information, these first *C*_A(E)_ values were included in the average to obtain the assumed *C*_A(E)_ values that comprised part of the measured value of *C*_1(E)_. In all cases, the contribution of *C*_A(E)_ to *C*_1(E)_ was sufficiently small (< 10 %) so that the effect on the comparison of the concentration ratios was somewhat insignificant.

Subsequent to the above analysis by NIST, Lab E independently re-evaluated the background ambient concentrations using a less conservative approach of excluding the first ambient values following a standard addition. The effect of these background choices on the (*C*_1(E)_ − *C*_A(E)_)/*C*_0_ results was negligible. The change in (*C*_1(E)_ − *C*_A(E)_)/*C*_0_ for any standard addition was typically less than 1 %, and ranged to 2.3 % in the worst case.

The comparisons of Table 3 indicate a remarkably excellent agreement between Lab E and NIST. The mean concentration ratios over the 53 comparisons is 0.97 (excluding ambient influence) and 0.94 (including ambient influences) with a standard deviation of the mean (*s*_m_) of approximately 2 % and a correlation coefficient *r* = 0.98. Considering the one standard deviation statistical “counting error” uncertainty in *C*_1(E)_ of several percent alone (see Fig. 5) and the 3 % to 4 % uncertainty in *C*_0_ (for a one standard deviation interval as given in the uncertainty analysis of Collé et al. [1]), the comparisons indicate that there are no statistically significant differences between the Lab E results and NIST standardized sample additions.

In addition, no significant systematic trends as a function of ^222^Rn concentration were evident. As seen in Fig. 7, the comparative concentrations scale in good agreement over the entire range. If anything, it appears, and surprisingly so, that the agreements (in terms of (*C*_1(E)_/*C*_0_) differences) are, in general, better at lower concentrations (4 Bq · m^−3^ to 20 Bq · m^−3^) than at the higher concentrations (25 Bq · m^−3^ to 35 Bq · m^−3^). This is not universally the case, however, if one considers the agreement on a relative basis, such as for standard addition #15 at the lowest introduced concentration. Additions #1, #2, and #3, and then #11 and #12 (see Fig. 7) are all in the general concentration range around 30 Bq · m^−3^ to 35 Bq · m^−3^; and for these cases, the concentration ratios *C*_1(E)_/*C*_0_ and (*C*_1(E)_ − *C*_1(A)_)/*C*_0_ appear to exhibit the greatest variations and deviations from unity. Nevertheless, the agreement is remarkably consistent across the entire concentration range.

One may note that the first measurements of *C*_1(E)_ for additions #1 and #2 appear to be abnormally high compared to the following three in each series. A question has arisen as to whether the NIST manifold was completely flushed of accumulated radon prior to the commencement of these additions. This could possibly account for the large initial values. Although the incomplete removal of previously accumulated radon can not be absolutely excluded as a possibility (particularly in the first few standard additions, e.g., #1, #2, and #3, when the NIST *persona grata* was somewhat unfamiliar with the test site’s experimental layout), it is not believed to have occurred. Also, these large positive deviations in additions #1 and #2 appear to be somewhat matched by the negative deviations of almost comparable magnitude of addition #11, for example, thereby suggesting that the exhibited deviations are statistical in nature.

One last observation may be made in regard to Fig. 8. The abrupt and large increase in the Bermudian ambient concentration following addition #15 was similarly exhibited in both the Lab F and Lab E data. The same trend is also seen in the data of Lab A and Lab D (Fig. 4). The introduced ^222^Rn activity concentration *C*_0_ for addition #15 was approximately 2.5 Bq · m^−3^. The observed increase in the natural ambient concentration following addition #15 went to experimentally-determined *C*_A_ levels of roughly 1 Bq · m^−3^ to 2 Bq · m^−3^. This was a surprisingly unexpected result (based on what the organizers led NIST to believe would be typical ambient concentrations). It was fortuitous in that it occurred at the conclusion of the standardized sample additions; and of good fortune in that it provided an almost complete overlap in the ^222^Rn activity concentrations covered in the intercomparison of Bermudian natural ambient air concentration levels *C*_A_ (< 0.01 Bq · m^−3^ to 2 Bq · m^−3^) and in the intercomparison of introduced *C*_0_ concentrations (≃ 2.5 Bq · m^−3^ to 35 Bq · m^−3^). Nature, and her attendant *Minerva*, cooperated.

### 4.2 Lab A Results

**Fig. 9:**
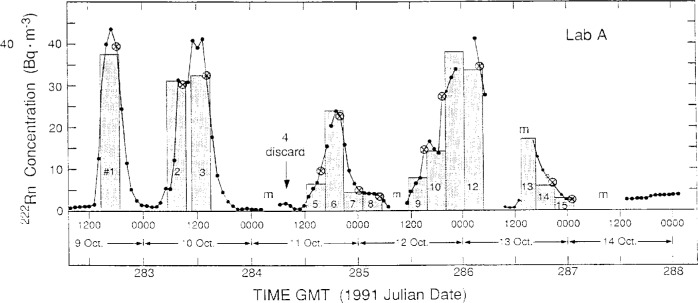
Lab A measurement results for the “smoothed,” hourly-averaged, mean ^222^Rn concentration *C*_1_ (plotted data points) compared to the standardized sample concentrations provided by NIST (shaded areas). Refer to the text for discussion.

The measurement results of Lab A for the mean ^222^Rn activity concentration *C*_1_ compared to the concentrations *C*_0_ provided by NIST for the 14 valid standardized sample additions are given in Table 4. Figure 9, which will greatly assist in the understanding of the analysis and interpretation of the comparisons, illustrates these data. The values of mean *C*_0_ averaged over the entire sample addition interval, as derived and given in Collé et al. [1], are shown as shaded regions. The plotted data points are the reported results of Lab A (see Fig. 3 and Appendix A).

It must be emphasized that the experimental design and protocol used for the standardized additions inherently were very inappropriate for evaluating the Lab A performance. The continuous and slow response of the Lab A instrument is highly suitable for continuous monitoring of slowly varying ambient concentrations. The artificially imposed ^222^Rn concentration step functions of the standard additions places the evaluation of Lab A at a serious disadvantage compared to automated instruments that operate with finite sample collection and measurement time intervals.

The analysis of the Lab A data is complicated in that the results *C*_1_ are hourly averages of continuously accumulated data “smoothed” by a time constant of 90 min. In effect, the average *C*_1_(*t_i_*) reported during some arbitrary time-interval period *t_i_* is influenced not only by the current concentration *C*_0_(*t_i_*) in the sampling stream line as it enters the Lab A delay tank, but is also influenced by the concentration *C*_0_(*t_i_* − *j*) that entered the delay tank in previous periods *t_i_* − *j* (where *j* = 1, 2, 3, …). By numerical integration, the concentration *C*_1_(*t_i_*) in period *t_i_* (excluding influences from ambient concentrations) may be expressed as

$$C_{1}\left(t_{i}\right)=\sum_{j=0}^{i}C_{0}\left(t_{i-j}\right)\alpha_{i-j}$$

where *α_i_*_−_ *_j_* is a removal function that takes into account the removal of ^222^Rn from the tank by decay and by ventilation during the period *t_i_*_−_ *_j_*. Thus, to obtain any given concentration *C*_1_(*t_i_*), the averaged measured response at a given time must be unfolded from all previous measurements of *C*_1_ during the influencing period. Using an assumed time constant of 90 min, as recommended by Lab A, this results in an ensemble of equations which must be simultaneously *χ*^2^-minimized and reduced to arrive at an unfolded set of uninfluenced *C*_1_ values. A few preliminary attempts were made to try to “unfold” this integral data set, but the attempts were soon abandoned because of the inherently large resulting uncertainties and failures of the minimizations to converge. It was apparent that the quality of the data could not possibly justify the numerical exercise.

An alternative analysis procedure was sought. Inspection of Fig. 9, and on consideration of the simple physics involved, one might have the following qualitative expectations: over the course of a 4 h sample addition with mean *C*_0_, the first hourly-averaged value *C*_1(A1)_ would be very low in comparison to *C*_0_; the next hourly-averaged value *C*_1(A2)_ would be greater than *C*_1(A1)_, but would probably still underestimate *C*_0_; the third value *C*_1(A3)_ would again be larger than *C*_1(A2)_, but might begin to approximate the range of *C*_0_; and the fourth value *C*1_(A4)_ might not be very different from *C*_1(A3)_. Thus, a simple and reasonable analysis approach would be to compare *C*_1(A3)_ and *C*_1(A4)_ to *C*_0_. In fact, this is the procedure adopted and presented in Table 4. To facilitate understanding the interpretations, the values of *C*_1(A4)_ are enlarged in the plot of Fig. 9. It could be argued that this approach is somewhat subjective; but, given the limitations of the adopted standard addition procedure for comparisons with this continuous measurement method, no other analysis procedure seemed feasible.

Even this approach is only partially applicable in the cases of adjoining standard sample addition intervals when the mean *C*_0_ is adjusted dramatically from one interval to the next. Influences from preceding concentrations *C*_0_ require the passage of at least approximately 4 h to 5 h intervals, as clearly seen in the return to ambient concentrations after additions #1 and #3 in Fig. 9. This is obviously affecting the results of addition #7 after the abrupt change from #6, and that of #14 following #13. This is the situation for decreasing step changes in *C*_0_. There is yet one more complicating feature in the data set of Lab A. It is apparent that the values of *C*_1(A4)_ for additions #5, #9, and #10, in which the following additions (#6, #10, and #11) are increasing step changes, are abnormally high. This strongly suggests a difference in timing between that reported for the Lab A data and that for the standard additions, as was discussed at considerable length in Collé et al. [1], in which the onset of activity concentration *C*_0_ for addition #*N* is reflected in the reported result *C*_1(A4)_ for addition #(*N* − 1). It, furthermore, calls into question the results of the comparison *C*_1(A4)_/*C*_0_ for additions #5, #9, and #10 in Table 4.

Figure 9 indicates that the effect of the previous concentration on *C*_1(A3)_ is not severe for an increasing step change. However, for a decreasing step, the peak contribution remaining at *C*_1(A3)_ after a decreasing step can cause a substantial over-estimate of *C*_0_. This is readily apparent for additions #7, #14, and #15. The relative magnitude of this over-response at *C*_1(A3)_ is roughly 10 %.

Interestingly, Lab A, in reporting its results, suggested using the value *C*_1(A2)_ for the intercomparison since they concluded that the maximum response *C*_1_(max) to a step change in concentration *C*_0_ would occur somewhat after 2 h following the time of change and that *C*_1(A2)_ would give values within a few percent of *C*_1_(max). However, the comparison *C*_1(A2)_/*C*_0_ was generally much worse than *C*_1(A3)_/*C*_0_ or *C*_1(A4)/_*C*_0_, particularly in the cases of decreasing step changes. The suggested timing difference also complicates even this conclusion.

Nonetheless, even with these inherent limitations, the results of Table 4 indicate a reasonably good agreement between Lab A and NIST. The mean value of *C*_1(A3)_/*C*_0_ is approximately 1.10 with a standard deviation of the mean (*s_m_*) of 4 % and a correlation coefficient of *r* = 0.975. One should not place too much emphasis on this 10 % agreement (or the even better agreement in *C*_1(A4)_/*C*_0_) since the actual magnitude could very well be somewhat coincidental considering the magnitudes of the statistical variations and the subjective aspects of the comparison. Figure 9, however, clearly illustrates the general tracking and reasonably good agreement of their “smoothed” *C*_1_ data with *C*_0_.

Lastly, it should be mentioned that considering the limitations of the experimental design for comparing the Lab A data to the standard additions, the quality of the data comparisons did not warrant attempts to account for contributions from ambient concentrations *C*_A_.

Lab A, following the original NIST analysis and report of the comparisons, suggested that the data set indeed warrants reconsideration. Lab A’s independent re-analysis involved using only *C*_1(A3)_ values, making background corrections using the assumed *C*_A(E)_ values of Table 3, and correcting these (*C*_1(A3)_ − *C*_A(E)_) values by “subtracting 10 % of the difference between the current and previous value when the previous value was larger, in order to account for the slow response of the detector.” Lab A thereby concluded that with the background subtraction and the correction on the basis of 10 % of the concentration change, comparison to NIST “substantially improves,” resulting in a mean corrected *C*_1(A3)_/*C*_0_ ratio of 1.027 with a relative standard deviation of the mean of 3.3 % based on 14 comparisons and with a correlation coefficient of 0.978. This may be compared to the NIST analysis of Table 4. It must also be emphasized that the 10 % correction suggested by Lab A was not known prior to the intercomparison, and was only derived as a result of the Lab A to NIST standard addition comparisons.

### 4.3 Lab D Results

The reported results for Lab D are summarized in Table 5 which contains the reported values of *C*_1(D)_ and estimated *C*_0(D)_ that were derived from their own assumed values for *C*_A_. The values for *C*_0_ provided by NIST were again taken from Collé et al. [1]. Comparisons of both *C*_1(D)_/*C*_0_ and *C*_0(D)_/*C*_0_ indicate a substantial systematic difference between the results of Lab D and that of NIST. The systematic proportional bias of approximately 0.37 in the concentration ratios (with *s_m_* = 4.5 % and *r* = 0.981) was invariant over the range of concentrations. This effect was attributed by Lab D to be a result of a calibration error introduced by using the assumed calibration factors provided by the manufacturer of a commercially-available, flow-through ^226^Ra calibration source that was used by Lab D for their calibrations. Deviations in possibly both the ^226^Ra activity content of commercial sources, as well as in the functional form for the available ^222^Rn concentration as a function of flow rate for flow-through sources, were demonstrated in Collé et al. [1].

The *C*_0_ values provided by NIST for Lab D and those provided for Lab E are, of course, highly correlated, being derived from slightly different subsets of the identical simultaneous flow-rate measurement data set. Therefore, it is not unexpected that the exclusively positive deviations from the mean concentration ratios as seen in additions #1, #2, #3, #9, and #12 (or the almost exclusively negative deviations from the mean as seen in additions #5, #6, #7, #8, and #15), although similarly occurring in the comparisons of the data for both Lab D and Lab E, are anything but random variations.

## 5. Intercomparison of Ambient Air Concentrations

For the intercomparison of mean ^222^Rn activity concentrations *C*_A_ in ambient Bermudian air among all four participating laboratories, the data set of Lab E was selected to provide the necessary normalization. It was chosen because it was not only the largest and most complete data set available, but it also seemed to be the data set whose *C*_A_ values were most in the midrange of all reported *C*_A_ values.

The comparisons were made by selecting pairs of the nearest adjacent values of *C*_A_, in terms of their reported midpoint times, for Lab E and for the other participating laboratory. The paired values [*C*_A(E)_ and *C*_A(LAB)_ where LAB = F,A,D] could then be used to form a set of comparison ratios *C*_A(LAB)_/*C*_A(E)_. For example, a *C*_A(F)_ value for Lab F whose midpoint time was 1700 hours GMT would be compared to the *C*_A(E)_ values at both 1630 and 1730 for Lab E. The Lab A *C*_A(A)_ and Lab E *C*_A(E)_ values typically had the same midpoint times and could be compared directly. No attempt was made to try to account for the “smoothing” effect in the Lab A data. The *C*_A(D)_ results of Lab D similarly were compared to the nearest *C*_A(E)_ values that were reported both before and after the midpoint time for the *C*_A(D)_ results. In no cases were any comparisons made for values whose midpoints were separated by more than 1.5 h. Also, every effort was made to avoid the selection of paired *C*_A(E)_ and *C*_A(LAB)_ values that might have been influenced by the introduced activity of the standardized sample additions. This pair-wise selection of reported values, then comprised the data sets that were analyzed over the course of the entire 2 week intercomparison period (excluding the intervals for the standardized sample additions).

Before addressing these pair-wise comparisons to the Lab E results, it should be understood that similar independent evaluations were also performed by making direct comparisons of *C*_A(A)_ to *C*_A(F)_, *C*_A(D)_ to *C*_A(F)_, and *C*_A(D)_ to *C*_A(A)_. In all cases, the results of the various comparison estimators (see Table 6) were statistically consistent and redundant with the results that follow.

### 5.1 Lab F to Lab E Comparison

**Fig. 10:**
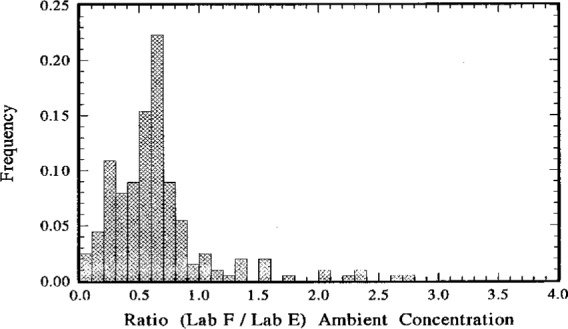
Distribution of the comparison ratios *C*_A(F)_/*C*_A(E)_ for 202 paired values of measurement results by Lab F and Lab E for the ^222^Rn activity concentration in ambient Bermudian air during the intercomparison period October 5–17, 1991.

**Fig. 11:**
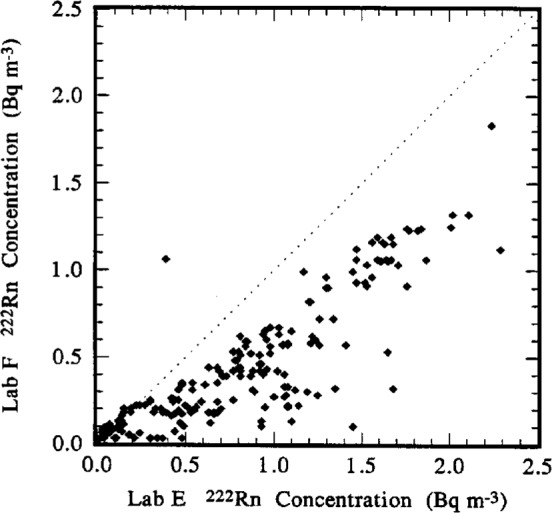
A scatter diagram of 202 paired values of *C*_A(F)_ and *C*_A(E)_ for the ^222^Rn activity concentration in ambient Bermudian air measured by Lab F and Lab E during the intercomparison.

For the comparison between Lab F and Lab E, the data set comprised 202 paired *C*_A(F)_ and *C*_A(E)_ values. The ratios *C*_A(F)_/*C*_A(E)_ ranged from a minimum of about 0.06 to a maximum of 2.7, with median and mean values of approximately 0.60 and 0.65, respectively. These descriptive statistical estimates are summarized in Table 6. A frequency distribution for the ratios *C*_A(F)_/*C*_A(E)_ is given in Fig. 10. Clearly, the measurements of *C*_A_ by Lab F are systematically low with respect to Lab E. This is even more apparent in the scatter diagram of Fig. 11 where the values of *C*_A(F)_ are plotted as a function of *C*_A(E)_. The dotted line in the figure is the “helping line” for when the comparison ratios are equal to unity, i.e., for the “ideality” *C*_A(F)_ = *C*_A(E)_. As indicated in the figure, the entire set of *C*_A(F)_ values are below those of *C*_A(E)_ for all *C*_A(E)_ values greater than 0.25 Bq · m^−3^. The one glaring datum exception (at *C*_A(E)_≃0.4 Bq · m^−3^ and *C*_A(F)_≃1 Bq · m^−3^) is obviously a fluke. The wide scatter of data in Fig. 11 is indicative of the inherent irreproducibility for these low-level measurements of ambient air, at least on a comparative basis for the results between laboratories. Recalling Fig. 5, the one standard deviation statistical “counting error” alone for *C*_A(E)_ = 0.5 Bq · m^−3^ to 1 Bq · m^−3^ was in the range of 10 % to 5 %. One of the participant laboratories suggested that the wide dispersion in *C*_A(F)_/*C*_A(E)_ ratios referred to above was not due to an inherent low-level measurement irreproducibility, but rather is a reflection of variations of the radon progeny to radon equilibrium ratios. The Lab F instrument at the relatively high ambient radon concentrations occurring at the time of the intercomparison had very good measurement repeatability (perhaps less than a few percent), yet the Lab F results would be strongly influenced by any changes in the equilibrium ratios which are in turn a function of condensation nuclei concentrations. Explicit correlations between radon concentrations and equilibrium ratios as a function of condensation nuclei concentrations, however, is beyond the scope of this work. Nevertheless, *C*_A(F)_ and *C*_A(E)_ are highly correlated with correlation coefficient *r* = 0.88, and as seen by the general trend of the data points in Fig. 11.

The results of a linear regression on sets of two measurement results which are estimates of the same quantity (e.g., in this case *C*_A(F)_ = *α* + *β C*_A(E)_ for the variables *C*_A(F)_ and *C*_A(E)_) that are estimating the “true” *C*_A_ are informative. The slope *β* (for intercept *α* ≃ 0) is often a better comparison estimator of the agreement between the two variables than is an estimator of central tendency (e.g., the mean or median) alone. This is particularly true when the variables have large statistical variability. Means of ratios (e.g., *C*_A(F)_/*C*_A(E)_) can be substantially influenced by even a few exceedingly high or exceedingly low values, and their exclusive use in comparisons can lead to seriously misleading or distorted results. (This point will be demonstrated later in the discussion of Lab D comparison results.)

The linear regression results for the Lab F comparison are given in Table 6. The intercept *α* is negligible, and the slope *β* = 0.59 is in very good agreement with both the mean (0.65) and median (0.60). One can therefore conclude that, for the purposes of this comparison, the measurement results of Lab F are somewhat systematically low with respect to those of Lab E by roughly 40 %. Excessive confidence should not be placed in this rough estimate because of the large data variability. Yet, it clearly is the general trend.

The Lab F measurement results might suggest that ^222^Rn was not in secular equilibrium with its daughters, and that the equilibrium ratios substantially differed from unity.

### 5.2 Lab A to Lab E Comparison

**Fig. 12:**
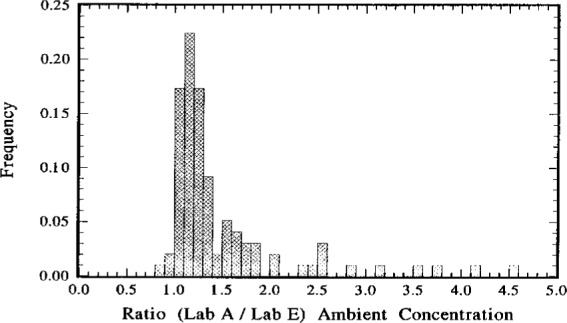
Distribution of the comparison ratios *C*_A(A)_/*C*_A(E)_ for 104 paired values of measurement results by Lab A and Lab E for the ^222^Rn activity concentration in ambient Bermudian air during the intercomparison period October 7–17, 1991.

**Fig. 13:**
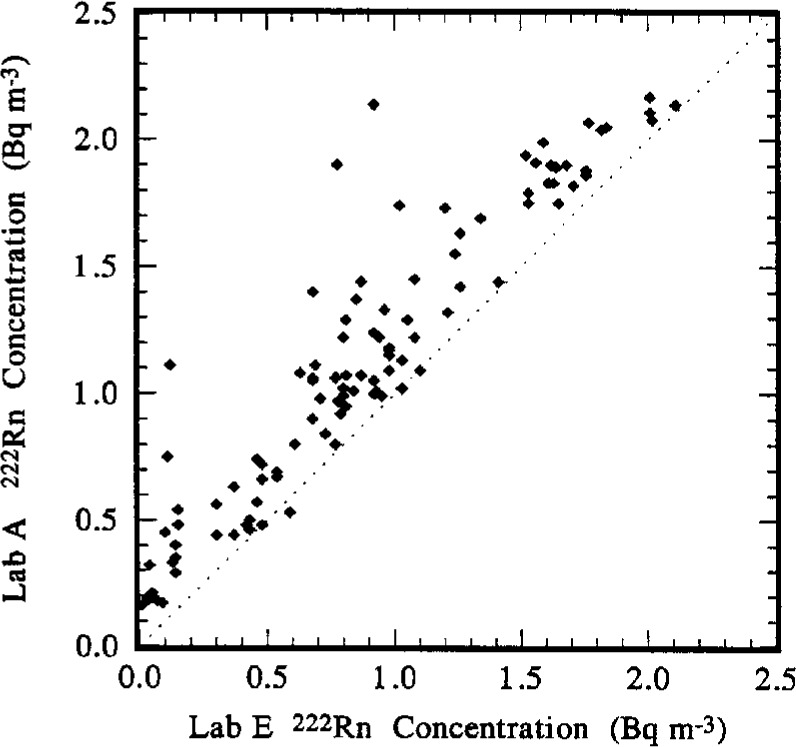
A scatter diagram of 104 paired values of *C*_A(A)_ and *C*_A(E)_ for the ^222^Rn activity concentration in ambient Bermudian air measured by Lab A and Lab E during the intercomparison.

Descriptive statistical estimates for the comparison between Lab A and Lab E are also given in Table 6. The comparison consisted of 104 paired *C*_A(A)_ and *C*_A(E)_ values. The ratios *C*_A(A)_/*C*_A(E)_ ranged from a minimum of approximately 0.9 to a maximum of 16. The median and mean values are 1.3 and 1.9, respectively, indicating that the Lab A values are in general systematically high with respect to those of Lab E. The frequency distribution of Fig. 12, and, even more so, the scatter diagram of *C*_A(A)_ versus *C*_A(E)_ of Fig. 13, confirm this generality. Considering that both Lab E and Lab A seemed to be in fairly good agreement with the standardized sample additions provided by NIST (Tables 2 and 3), their apparent systematic differences for the comparison of ambient air concentrations may seen dichotomous. Part of this may be understandable by inspection of Fig. 13 and the results of the linear regression in Table 6. Note that the slope *β* is very nearly equal to unity which in itself would indicate good agreement with Lab E (and NIST by inference). However, the intercept of the regression is substantial, *α* = 0.27, which implies a uniformly offsetting bias of about 0.3 Bq · m^−3^ that is more significant at low concentrations (e.g., ambient air) than at higher concentrations (e.g., in the standardized sample additions). Possible reasons for this non-zero offset in terms of the Lab A and Lab E instrumentation are unknown. Both the generally higher results of Lab A and the non-zero offset in comparison to Lab E may, of course, be due to unaccounted thoron (^220^Rn) background contributions with the Lab A results. Equally possible, the apparent offset may not necessarily be attributable to an offset by the Lab A results, but rather may be due to a negative offset in the Lab E results due to an over-subtraction of background by Lab E. The latter possibility is supported in part by comparisons of the Lab A to Lab D results (after invoking a 65 % to 70 % correction to the Lab D results as concluded from the comparisons to standardized additions as given in Sec. 4.3).

### 5.3 Lab D to Lab E Comparison

**Fig. 14:**
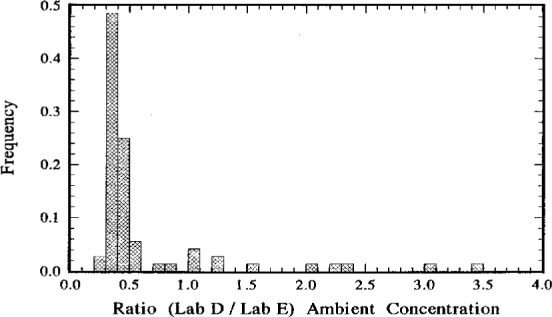
Distribution of the comparison ratios *C*_A(D)_/*C*_A(E)_ for 72 paired values of measurement results by Lab D and Lab E for the ^222^Rn activity concentration in ambient Bermudian air during the intercomparison period October 7–17, 1991.

**Fig. 15:**
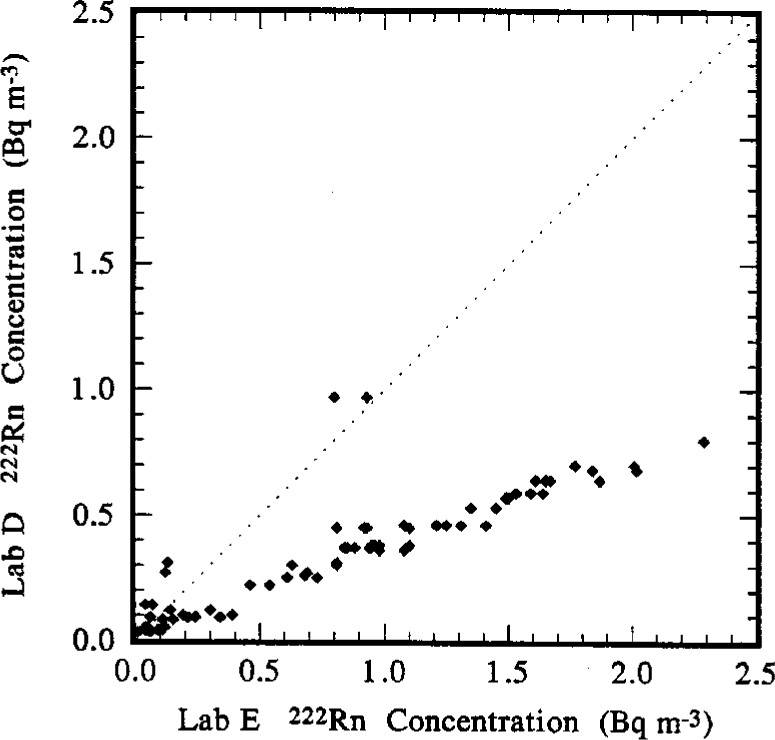
A scatter diagram of 72 paired values of *C*_A(D)_ and *C*_A(E)_ for the ^222^Rn activity concentration in ambient Bermudian air measured by Lab D and Lab E during the intercomparison.

As for the previous comparisons, the comparison between Lab D and Lab E is summarized in Table 6 and in the frequency distribution and scatter diagram of Figs. 14 and 15. In this case, 72 paired *C*_A(D)_ and *C*_A(E)_ values were compared yielding for the ratio *C*_A(D)_/*C*_A(E)_ a minimum of 0.3, a maximum of 3.5, a median of 0.4, and a mean of 0.63. Obviously, the Lab D results are systematically low with respect to the Lab E measurements. The results are entirely consistent with the comparisons of the standardized sample additions (Table 5). The linear regression slope *β* (0.33) is a much better indicator than the mean (0.63) and is in very good agreement with the mean ratios (0.37 and 0.36) given in Table 5.

The intercept of the regression is 0.076 Bq · m^−3^. If one corrects this value by a reciprocal correction factor of 0.361 obtained from the results of the standardized addition comparisons (Table 5), then one obtains a corrected value of 0.21 Bq · m^−3^ for the Lab D to Lab E regression intercept. This value is remarkably close to that obtained for the Lab A to Lab E comparison intercept (0.27 Bq · m^−3^) and adds considerable credence to the possibility that the Lab E results have a negative bias due to over correcting for background.

One may conclude, nevertheless, that for the purpose of this intercomparison, the measurement results of Lab D are proportionately biased and systematically low with respect to the measurements of Lab E by roughly 65 % to 70 %, irrespective of possible systematic background-correction offset biases.

## 6. Discussion of Findings and Summary Thoughts

The results of the intercomparison itself are nearly self-evident on examination of the summaries in Tables 3, 4, and 5 for standardized sample additions, and Table 6 for the ambient concentrations.

Lab E and Lab A were in excellent agreement with the NIST additions. The vast majority of reported values for both laboratories, over the entire 2.5 Bq · m^−3^ to 35 Bq · m^−3^ concentration range, were within the 6 % to 13 % relative 3 standard deviation uncertainty interval associated with the NIST additions. The Lab D results for the standardized additions were obviously proportionately biased by a mean factor of about 0.36, which may be attributable to an instrument calibration error. The Lab D reported concentrations, on correcting all of Lab D’s values by this common factor, are then in good agreement with those of both Lab E and Lab A. Further, the Lab E data, Lab A data, and corrected Lab D data track the fluctuations of radon concentrations very well over the entire range of concentrations even though the number of data values from the three laboratories was substantially different.

The intercomparison of reported ambient concentration values was performed by normalizations to the Lab E data. The results were statistically invariant of this arbitrary choice—normalizing to any other laboratory set results in redundant and statistically equivalent comparison estimators, e.g., means, linear regression slopes, etc. (see Table 6). These ambient concentration results reinforced the findings obtained from the standardized sample additions. The slope (*β*) of a linear regression of the reported ambient concentrations from the two laboratories under comparison was considered the best available comparison estimator. Concentration pairs selected for the comparison regressions were the measurement values nearest to each other in terms of their midpoint times. For comparison of Lab A to Lab E, *β* = 0.97 ± 0.11 which again is indicative of the good agreement between these two laboratories results. This uncertainty interval and all of the uncertainty intervals that follow are assumed to correspond to three standard deviations and are assumed to provide an uncertainty interval having a high level of confidence of roughly 95 % to 99 %. For the Lab D to Lab E comparison, *β* = 0.33 ± 0.06, which exhibits a proportional bias of the same magnitude found in the comparison to standardized sample additions. Again, if one evaluates the Lab A data, Lab E data, and corrected Lab D data (after correcting all of the Lab D values by a common reciprocal correction factor of 0.36), one finds that all three laboratories are in good agreement in tracking changes in the ambient concentrations from the relatively high 2 Bq · m^−3^ ambient levels down to concentrations of a few hundredths of a Bq · m^−3^. Differences were greatest at the very lowest ambient concentrations because of an apparent offset in the regressions among the laboratories (refer to the discussions in Sec. 5). The offset (regression intercept *α*) between Lab A and Lab E was *α* = (0.27 ± 0.11) Bq · ^−3^, which has been suggested to be attributable to either an unaccounted ^220^Rn (thoron) contamination background for Lab A or an over-corrected background for Lab E. The offset between Lab D and Lab E was nearly negligible, *α* = (0.076 ± 0.068) Bq · m^−3^. However, if one invokes the same correction as before to the Lab D data, then the corrected intercept *α* = (0.23 ± 0.22) Bq · m^−3^ would seem to support the latter over-corrected background argument. The magnitude of the uncertainty intervals for the intercept values, however, does not make the argument compelling. For the Lab F to Lab E comparison, *β* = 0.59 ± 0.07, indicating an approximate 40 % disagreement which, as noted, was surprising compared to the reported results of previous intercomparisons. This difference is equally manifest in direct comparisons between the Lab F and Lab A measurement data and in direct comparisons between Lab F data and corrected Lab D data. The Lab F to Lab E intercept, *α* = (− 0.04 ± 0.07) Bq · m^−3^, is truly negligible. This result does not support the previously observed offset between Lab A and Lab E even if the Lab F data is renormalized (i.e., “corrected”) by the reciprocal of the observed *β* between Lab F and Lab E. Throughout the course of the ambient concentration intercomparison period, over all concentration ranges, the Lab F data also appropriately scaled (by a factor of roughly 0.6) over all concentration ranges with respect to the Lab A data and similarly scaled (by a factor of roughly 1.8) with respect to the Lab D data.

A considerable number of additional statistical analyses beyond those reported here were performed on the data sets. These included: (1) sequential time analyses to determine if there were any time dependencies or correlations in the observed measurement differences between the participating laboratories or with the NIST standardized sample additions; (2) regression analyses between the results of each participating laboratory and the NIST additions; (3) regression analyses between every combination of participating laboratory pair; (4) *χ* ^2^-tests for all regressions and comparison frequency distributions; (5) divisions of the ambient concentration comparison data for the participating laboratory pairs first into halves (and then into thirds), and testing the resulting subsets of data for differences in the various means using *t*-tests, and for homogeneity in the various subset sample means and variances using *χ*^2^- and *F*-tests; and (6) sequential two-variable analysis-of-variance (ANOVA) techniques for differences in similarly constructed subset means and variances. The results of these analyses were not reported in an attempt at brevity in an already too-lengthy paper, and since they added nothing to the analyses that were reported nor to the findings and conclusions.

In order to maintain the integrity of the intercomparison, Dr. R. Collé, representing NIST, retained overriding authority among the co-authors with regard to the statements of the results and the conclusions as reported herein. The data interpretations, design of the evaluation procedures, and statistical analyses are his, and his alone. Each of the participating laboratories provided supplemental information, such as descriptions of their respective instruments and measurement methodologies, contributed to the discussion, and had an opportunity to comment on the NIST analyses.

The design of the intercomparison was as near as the investigators could come to conducting a “blind” comparative exercise, as described in the proposal submitted by the organizers of the intercomparison, the Drexel University investigators, to the National Science Foundation. The exercise was “blind” in several regards: (1) the standard sample additions provided by NIST were introduced with undisclosed ^222^Rn activity concentrations, and the NIST results were not disclosed and released until all of the participating laboratories had provided their respective measurement data; (2) the timing and duration of the NIST standard sample additions were also largely unknown to the participating laboratories; (3) NIST participation required that once the participating laboratories reported their measurement values, the data would be analyzed and reported without subsequent modifications or corrections to the originally reported values; (4) although NIST knew the approximate activity concentrations at the time of the introduction of the standardized sample additions, the final mean activity concentrations provided to each participating laboratory during their respective sampling intervals were not known by NIST until after reduction of the extensive flow rate data base; and (5) NIST provided the standardized sample additions largely in complete ignorance of the underlying ^222^Rn ambient concentration at the time of the additions.

It must be emphasized that this intercomparison was not intended to critique or evaluate the various instruments or measurement methodologies in terms of their advantages, disadvantages, or suitability for performing continuous ^222^Rn monitoring in marine atmospheres. The sole intent was, as stated, to provide an unbiased and refereed intercomparison of ^222^Rn activity concentration measurements made by four laboratories, and to provide three of these laboratories, under somewhat inappropriate and limiting experimental test conditions for at least one laboratory, with introduced samples containing known, but undisclosed (i.e., “blind”) ^222^Rn activity concentrations that could be referenced to U.S. national standards. To wit, for purposes of this intercomparison exercise, the various instruments and measurement methodologies were viewed much as how the diversity of prevailing religious modes of worship was regarded by magistrates in the late Roman empire: all equally true; all equally false; all equally useful [14].

This intercomparison was not without imposed limitations. Although all of the principal measurement methodologies used to continuously monitor marine air masses—where typical concentrations are much lower than found in continental air masses and where humidity levels vary—were represented by the participating laboratories, funding and space limitations of the experimental site could not accommodate other groups making atmospheric radon measurements. It is arguable that the intercomparison exercise was too short in time duration. Excluding the period for the intercomparison of standardized sample additions, the time for intercomparison of ambient air concentrations was of the order of 2 weeks, and even this period was not continuous. Without question, continuous intercomparison measurements over longer time intervals, two or more uninterrupted weeks or even months, would have been much better. Equally, it would have been more useful to conduct correlations with meteorological data and with ^222^Rn progeny measurements and equilibrium ratios. These correlations would have been useful to discriminate the air masses’ origin, to know if they are of direct continental origin, or marine origin, or mixed. In this way, it might have been possible to discern if the apparent discrepancies among some of the participating laboratories were different in relation to differing meteorological situations. The differences between the measurements of Lab A and Lab E, for example, were not apparent in previous intercomparisons [11]. These kinds of efforts, however, were beyond the scope of this work.

## 7. Conclusions

This exercise was unique among other environmental intercomparisons, and it fulfilled two major objectives.

Firstly, this work provided an unbiased, refereed basis for comparing the measurement results and performance of four principal instruments (as employed by four different laboratories) that are used to measure ^222^Rn activity concentrations for marine-atmospheric studies. Collectively, these instruments and laboratories represent those responsible for a significant fraction of the atmospheric ^222^Rn measurements made over the past decade. The intercomparison utilized a common standardized, *in situ*, reference basis that could be directly related to U.S. national, and internationally recognized, ^226^Ra and ^222^Rn standards. The findings of the intercomparison may assist various users (e.g., those in the global modelling community) in applying the available and future ^222^Rn measurement data bases in a more reliable and effective manner.

Secondly, the work went beyond serving the needs of this particular intercomparison. It also demonstrated the broader utility of the calibration protocol and the methodology for the standardized sample additions that were developed for it [1]. Most environmental measurement intercomparisons of field instruments in actual use merely rely on evaluating the relative performance of the participants, or some comparison to the pooled results. This exercise demonstrated, for the very first time, the capability of providing a standardized reference basis even for such low-level, field-measurement intercomparisons. The developed methodologies presented here could, of course, be adopted with slight modifications to cover other ^222^Rn concentration ranges and other applications, and could be employed in many other types of ^222^Rn environmental, field-measurement intercomparisons.

## Figures and Tables

**Table 1:** Comparative summary of the instruments and measurement methodologies used by the participating laboratories

	Lab E	Lab A	Lab D	Lab F
Instrument/methodology	Automated, intermittent 2-filter tube method; 500 L decay chamber; moveable filter ribbon	Continuous 2-filter tube method; 200 L drum followed by 50 min residence in 1000 L chamber with introduced condensation nuclei (controlled and calibrated)	Automated, intermittent, cryogenic separation of ^222^Rn from air on charcoal; transfer to counting cells	Automated, intermittent collection of ^222^Rn progeny on filters; infer ^222^Rn with equilibrium ratio assumptions; rotating tray of 12 filters
Detector	ZnS(Ag) scintillator for α detection	ZnS(Ag) scintillator for α detection	6 Lucas-type ZnS(Ag) scintillation cells for α detection	α-sensitive scintillator
Sampling flow rate	350 L · min^−1^ to 400 L · min^−1^	40 L · min^−1^	28 L · min^−1^	200 L · min^−1^
Sampling/measurement time	1 h sample collection on moveable filter; 1 h measurement of filter	Continuous measurement “smoothed” by 90 min time constant; 1 h measurement intervals reported; 30 min filter accumulation	2 h sample collection every 4.5 h; multiple hour measurement after secular equilibrium	2 h sample collection on moveable filter; 2 h measurement of filter
Sensitivity (efficiency or detection limit)	0.1 · s^−1^ per Bq · m^−3^; LLD = 0.12 Bq · m^−3^ equivalent ^222^Rn conc.	0.25 · s^−1^ per Bq · m^−3^; detection limit 0.01 Bq · m^−3^ equivalent ^222^Rn conc.	LLD = 0.004 Bq · m^−3^ equivalent ^222^Rn conc.	25 % α counting efficiency; detection limit 0.0004 Bq · m^−3^ equivalent ^222^Rn conc.
Background, counting rate	Measurement every 30 h; 0.02 · s^−1^; known thorium contamination	< 0.02 Bq · m^−3^ equivalent ^222^Rn conc.	2 h background counts on each cell before each measurement; 0.07 Bq to 0.17 Bq equiv. ^222^Rn	0.0006 · s^−1^
Calibration	In EML Radon, Thoron, and Progeny Exposure Facility	Internal, solid ^226^Ra/^222^Rn reference standard; and with standardized ^222^Rn injection at ANSTO	Internal, commercial (Pylon) ^226^Ra/^222^Rn reference standard	α efficiency relative to β^−^ counting of ^222^Rn progeny in equilibrium on same kind of filter
Uncertainty	Overall calibration uncertainty < 10 %; see Fig. 2 and discussion for statistical (Poisson) counting precision	Calibration uncertainty ± 15 %; see Fig. 3 and discussion for statistical (Poisson) counting uncertainty.	± 3.4 % to ± 3.9 % over intercomparison conc. range; measurement uncertainty ± 2.8 % over range (see disc. Sec. 3)	± 2 % α-counting efficiency uncertainty; overall estimated uncertainty ± 20 % in range 0.07 Bq · m^−3^ to 0.02 Bq · m^−3^
References	[2–5]	[6–9]	[10]	[11]

**Table 2:** NIST standardized sample additions

	Approximate time		Nominal range for the ^222^Rn activity concentration (Bq · m^−3^)
No.	1991 Date	Hours (GMT)	
1	9 October	1900–2300	33 to 37
2	10 October	0900–1300	28 to 30
3	10 October	1400–1800	29 to 31
4	11 October	0900–1300	discarded
5	11 October	1400–1800	5.3 to 6.2
6	11 October	1800–2200	20 to 23
7	11–12 October	2200–0200	3.9 to 5.0
8	12 October	0200–0600	3.8 to 4.1
9	12 October	1200–1600	7.2 to 7.9
10	12 October	1600–2000	12 to 14
11	12 October	2000–0000	32 to 36
12	13 October	0000–0400	30 to 33
13	13 October	1200–1500	15 to 16
14	13 October	1500–1900	5.4 to 5.8
15	13 October	1900–2300	2.4 to 2.6

**Table 3:** Lab E reported results for the standardized additions

No.	^222^Rn concentration (Bq · m^−3^)			Concentration ratios	
	Lab E		NIST Stand. Add. *C*_0_	*C*_1(E)_/*C*_0_	*C*_1(E)_ − *C*_A(E)_
	*C*_1(E)_	Assumed *C*_A(E)_			*C*_0_
1	42.35	1.4	35.81	1.183	1.144
	34.70		33.55	1.034	0.993
	33.45		32.87	1.018	0.975
	37.29		37.07	1.006	0.968
2	41.87	1.5	29.09	1.439	1.388
	33.20		27.92	1.189	1.135
	33.55		28.82	1.162	1.112
	35.96		30.48	1.180	1.131
3	32.46	1.5	29.81	1.089	1.039
	34.39		28.59	1.200	1.150
	34.66		30.44	1.139	1.089
	36.26		31.23	1.161	1.113
5	4.90	0.07	5.349	0.916	0.903
	—		5.467	—	—
	5.39		5.787	0.931	0.919
	4.98		5.782	0.861	0.849
6	19.52	0.07	21.94	0.890	0.887
	18.88		20.43	0.924	0.921
	19.83		21.74	0.912	0.909
	21.22		21.87	0.970	0.967
7	5.13	0.07	5.013	1.023	1.009
	3.40		4.141	0.821	0.804
	3.43		3.946	0.869	0.852
	3.46		4.143	0.835	0.818
8	3.41	0.07	4.046	0.843	0.826
	3.23		3.978	0.812	0.794
	3.14		3.833	0.819	0.801
	3.20		3.898	0.821	0.803
9	7.12	0.15	7.350	0.969	0.948
	7.18		7.181	1.000	0.979
	7.75		7.524	1.030	1.010
	7.90		7.885	1.002	0.983
10	12.91	0.15	13.41	0.963	0.952
	11.98		12.39	0.967	0.955
	11.92		12.67	0.941	0.929
	12.78		13.70	0.933	0.922
11	30.41	0.15	36.25	0.839	0.835
	28.42		34.46	0.825	0.820
	28.84		32.47	0.888	0.884
	30.29		33.49	0.904	0.900
12	—	0.15	33.48	—	—
	31.69		31.71	0.999	0.995
	27.80		28.30	0.982	0.977
	31.28		29.64	1.055	1.050
13	13.64	0.17	14.79	0.922	0.911
	15.09		15.68	0.962	0.952
	15.63		15.66	0.998	0.987
14	5.62	0.17	5.770	0.974	0.945
	5.28		5.422	0.974	0.942
	5.29		5.671	0.933	0.903
	5.87		5.811	1.010	0.981
15	2.18	0.17	2.556	0.853	0.786
	1.91		2.371	0.806	0.734
	1.79		2.383	0.751	0.680
	1.92		2.465	0.779	0.710

			Number	53	53
			Mean	0.968	0.943
			*s_m_* (%)^a^	1.9	1.8
			*r*^b^	0.976	0.980

a Relative standard deviation of mean.

b Correlation coefficient.

**Table 4:** Lab A reported results for the standard additions

No.	^222^Rn concentration (Bq m^−3^)			Concentration ratios	
	Lab A		NIST Std.		
	*C*_1(A3)_^a^	*C*_1(A4)_^b^	*C*_0_	*C*_1(A3)_/*C*_0_	*C*_1(A4)_/*C*_0_
1	40.66	36.38	34.91	1.17	1.04
2	29.66	28.02	29.20	1.02	0.96
3	38.43	30.21	30.11	1.28	1.00
5	6.52	9.32	6.09	1.07	(1.53)
6	22.36	21.3	22.53	0.99	0.95
7	6.34	4.85	4.35	1.46	1.12
8	4.02	3.39	4.06	0.99	0.83
9	7.46	13.95	7.68	0.97	(1.82)
10	13.17	25.39	13.27	0.99	(1.91)
11	31.29		35.01	0.89	
12	38.69	31.83	31.06	1.25	1.02
13	15.47		15.78	0.98	
14	7.58	6.62	5.80	1.31	1.14
15	2.67	2.77	2.52	1.06	1.10
			Number	14	9
			Mean (*m*)	1.101	1.018
			*s_m_* (%)^b^	4.0	3.2
			*r*^c^	0.975	0.998

a The reported concentrations for the third [*C*_1(A3)_] and fourth [*C*_1(A4)_] hourly sampling (measurement) intervals after the start of the standardized sample additions.

b Relative standard deviation of the mean.

c Correlation coefficient.

**Table 5:** Lab D reported results for the standard additions

No.	^222^Rn concentration (Bq m^−3^)			Concentration ratios	
	Lab D		NIST Std.	*C*_1(D)_/*C*_0_	*C*_0(D)_/*C*_0_
	*C*_1(D)_^a^	*C*_0(D)_^b^	*C*_0_		
1	14.15	13.83	33.43	0.423	0.414
2	12.12	11.67	28.41	0.426	0.411
3	12.57	12.11	28.93	0.434	0.419
5	2.13	2.10	6.22	0.343	0.337
6	7.22	7.18	21.76	0.332	0.330
7	1.31	1.27	4.08	0.320	0.312
8	1.20	1.16	3.98	0.301	0.292
9	3.64	3.59	7.35	0.496	0.488
10	4.34	4.28	12.84	0.338	0.334
11	10.99	10.93	33.36	0.329	0.328
12	13.22	13.16	29.20	0.453	0.451
13	3.02	2.93	(8.43)	(0.358)	(0.347)
14	2.00	1.91	5.54	0.362	0.345
15	0.72	0.63	2.48	0.289	0.254
			Number	14	14
			Mean (*m*)	0.372	0.361
			*s_m_* (%)^c^	4.5	4.8
			*r*^d^	0.980	0.982

a The reported total concentration (as observed) due to both the ambient air and standard addition contributions. The reported relative uncertainties ranged from 3.4 % to 3.9 %.

b The reported net concentration for the contribution due only to the standard addition. It was calculated by subtracting the ambient concentration (as assumed by Lab D) obtained from adjacent measurement intervals. The reported relative uncertainties ranged from 3.4 % to 4.1 %.

c Relative standard deviation of the mean.

d Correlation coefficient

**Table 6:** Results for the intercomparison of ^222^Rn activity concentrations in ambient Bermudian air (October 5–17, 1991) among the participating laboratories (normalized to Lab E)

Comparison estimator	^222^Rn ambient concentration ratios			
	*C*_A(F)_/*C*_A(E)_	*C*_A(A)_/*C*_A(E)_	*C*_A(D)_/*C*_A(E)_	“Ideality”
Number of comparisons	202	104	72	
Minimum	0.063	0.898	0.256	1
Maximum	2.72	16.0	3.50	1
Median	0.600	1.251	0.399	1
Mean (m)	0.649	1.908	0.628	1
*s_m_* (%)^a^	4.7	10.3	11.5	
Correlation coefficient (*r*)^b^	0.877	0.931	0.878	1
Linear regression^c^ intercept (*α*, in Bq · m^−3^)	−0.037	0.270	0.076	0
*s_α_*(%)^d^	61	14	30	
Linear regression^c^ slope (*β*)	0.592	0.974	0.331	1
*s_β_*(%)^d^	3.9	3.9	6.5	

a Relative standard deviation of the mean.

b Correlation coefficient for the relative mutual dependence of variables *C*_A(LAB)_ and *C*_A(E)_, where LAB = F, A, D.

c For the regression function *C*_A(LAB)_ = *α* + *β C*_A(E)_, where LAB = F, A, D.

d Relative standard deviation of the mean for the regression coefficients.

## 8. Appendix A. Reported Measurement Results of the Participating Laboratories

The complete data set of measurement results from the participating laboratories, as reported by them, over the intercomparison period October 5–17, 1991 is given in Table A. The table includes reported values of the mean ^222^Rn activity concentrations over their individual sampling/measurement intervals for both the standardized sample additions (*C*_1_) and for the ambient air measurements (*C*_A_). The times (Table A, column 1) correspond to approximate mid-point times for each sampling/measurement interval. The concentrations reported by each laboratory were converted to common units of Bq · m^−3^ for comparison. The results for Lab F represent only measurement of (*C*_A_).

**Table A:** Reported measurement results of the participating laboratories

Time^a^ October, 1991 (GMT)		^222^Rn concentration (Bq · m^−3^)				Std. add. no.
Day	Hour	Lab F^b^	Lab A^c^	Lab D^d^	Lab E^e^	
5	1330				0.54	
	1430				0.43	
	1500	0.259				
	1530				0.42	
	1630				0.55	
	1700	0.189				
	1730				0.46	
	1830				0.44	
	1900	0.170				
	1930				0.48	
	2030				0.48	
	2100	0.171				
	2230				0.66	
	2300	[0.18]^f^				
	2330				0.66	
6	0030				0.32	
	0100	0.178				
	0130				0.53	
	0230				0.52	
	0300	0.203				
	0330				0.70	
	0430				1.12	
	0500	0.309				
	0530				0.88	
	0630				1.19	
	0700	[0.296]				
	0730				0.89	
	0830				1.08	
	0900	[0.326]				
	0930				1.06	
	1100	[0.266]				
	1130				1.00	
	1230				0.59	
	1330				0.65	
	1430				0.53	
	1530				0.52	
	1630				0.52	
	1730				0.49	
	1830				0.47	
	1900	0.122				
	1930				0.64	
	2030				0.68	
	2100	0.180				
	2130				0.63	
	2230				0.95	
	2300	[0.212]				
	2330				1.08	
7	0013			0.46		
	0030				1.25	
	0100	0.278				
	0130				1.07	
	0230				1.08	
	0300	[0.215]				
	0330				1.14	
	0430				1.10	
	0441			0.45		
	0500	0.128				
	0530				0.93	
	0700	0.098				
	0730				0.49	
	0830				0.24	
	0900	0.064				
	0911			0.09		
	0930				0.06	
	1030				0.44	
	1100	0.067				
	1130				0.14	
	1230				0.04	
	1300	0.056				
	1330				0.07	
	1341			0.14		
	1500	0.063				
	1630				0.04	
	1700	0.085				
	1730				0.11	
	1813			0.05		
	1830				0.12	
	1900	0.081				
	1930				0.06	
	2030				0.06	
	2100	0.080				
	2130				0.03	
	2230		0.32		0.04	
	2246			0.05		
	2300	0.071				
	2330				0.05	
8	0030		0.19		0.03	
	0100	0.074				
	0130		0.18		0.03	
	0230		0.17		0.09	
	0300	0.082				
	0324			0.04		
	0330		0.17		0.09	
	0430		0.16		0.01	
	0500	0.073				
	0530		0.19		0.05	
	0630		0.18		0.07	
	0700	0.106				
	0730		0.33		0.13	
	0758			0.31		
	0830				0.81	
	0900	0.623				
	0930				1.22	
	1030				1.30	
	1100	[0.983]*^g^				
	1130				1.56	
	1230				1.50	
	1234			0.57		
	1330				1.49	
	1430				1.68	
	1500	0.321				
	1630				1.35	
	1700	0.100				
	1709			0.53		
	1730				1.45	
	1830				1.29	
	1900	0.0989				
	1930				1.17	
	2030				1.30	
	2100	0.901				
	2130				1.31	
	2145					
	2230				1.21	
	2300	0.823				
	2330				1.20	
9	0030		1.73			
	0100	0.692				
	0130		1.59			
	0226			0.55		
	0230		1.54			
	0300	0.642				
	0330		1.52			
	0430		1.46			
	0500	0.548				
	0530		1.40			
	0630		1.24			
	0700	0.511				
	0705			0.30		
	0730		1.15			
	0830		1.08			
	0900	0.390				
	0930		1.02			
	1100	0.348				
	1145			0.26		
	1230		0.90		0.68	
	1330		1.05		0.68	
	1430		1.06			
	1500	0.440				
	1530		1.11		0.12	
	1625			0.27		
	1630		1.11		0.69	
	1700	0.418				
	1730		1.90		0.77	
	1830		12.18		1.65	
	1900	0.531				
	1930		*28.28*^h^		*42.35*	#1
	2030		*37.05*		*34.70*	#1
	2059			*14.15*		#1
	2100	0.495				
	2130		*40.66*		*33.45*	#1
	2230		*36.38*		*37.29*	#1
	2300	0.480				
	2330		22.96		3.29	
10	0030		11.07		1.06	
	0100	0.404				
	0130		5.17		0.94	
	0134			0.37		
	0230		2.71		0.88	
	0300	0.417				
	0330		1.74		1.02	
	0430		1.44		0.87	
	0500	0.393				
	0530		1.29		0.81	
	0618			0.45		
	0630		1.24			
	0700	0.420				
	0730		2.14		0.92	
	0830		5.32		*0.96*	
	0900	0.426				
	0930		*5.11*		*41.87*	#2
	1030		*11.52*		*33.20*	#2
	1053			*12.12*		#2
	1100	0.390				
	1130		*29.66*		*33.55*	#2
	1230		*28.02*		*35.96*	#2
	1300	0.284				
	1330		28.90		2.51	
	1430		*38.38*		*32.46*	#3
	1500	0.282				
	1526			*12.57*		#3
	1530		*36.53*		*34.39*	#3
	1630		*38.43*		*34.66*	#3
	1700	0.254				
	1730		*30.21*		*36.26*	#3
	1830		16.47		2.31	
	1900	0.212				
	1930		8.44		0.56	
	2030		4.44		2.39	
	2100	[0.215]*				
	2130		2.44		0.50	
	2230		1.40		0.68	
	2300	[0.243]*				
	2330		0.53		0.59	
11	0030		0.74		0.46	
	0100	0.250				
	0130		0.80		0.77	
	0230		0.56		0.30	
	0300	0.034				
	0330		0.48		0.48	
	0430		0.63		0.37	
	0500	0.030				
	0530				0.34	
	0610			0.09		
	0630				0.21	
	0700	0.026				
	0730				0.10	
	0830		1.59		0.01	
	0900	[0.015]*				
	0930		*2.00*		*4.37*	#4 (discard)
	1030		1.44		0.04	
	1036			0.04		
	1100	0.026				
	1130		0.75		0.11	
	1230		0.45		0.10	
	1300	0.028				
	1330		1.12		0.02	
	1430		*3.32*		*4.90*	#5
	1500	0.025				
	1505			*2.13*		#5
	1530		*5.25*			#5
	1630		*6.52*		*5.39*	#5
	1700	0.030				
	1730		*9.32*		*4.98*	#5
	1830		*14.89*		*19.52*	#6
	1900	0.031				
	1930		*19.23*		*18.88*	#6
	1935			*7.22*		#6
	2030		*22.36*		*19.83*	#6
	2100	0.027				
	2130		*21.32*		*21.22*	#6
	2230		*14.89*		*5.13*	#7
	2300	0.024				
	2330		*9.23*		*3.4*0	#7
12	0005			*1.31*		#7
	0030		*6.34*		*3.43*	#7
	0100	0.037				
	0130		*4.85*		*3.46*	#7
	0230		*4.39*		*3.41*	#8
	0300	0.033				
	0330		*4.24*		*3.23*	#8
	0430		*4.02*		*3.14*	#8
	0435			*1.20*		#8
	0500	0.034				
	0530		*3.39*		*3.20*	#8
	0630		2.12		0.18	
	0700	0.046				
	0730		1.04		−0.03	
	0830				0.01	
	0900	0.065				
	0904			0.03		
	0930				0.06	
	1030				0.03	
	1100	0.059				
	1130		1.98		−0.03	
	1230		*4.83*		*7.12*	#9
	1300	0.081				
	1330		*6.06*		*7.18*	#9
	1332			*3.64*		#9
	1430		*7.46*		*7.75*	#9
	1500	0.072				
	1530		*13.95*		*7.90*	#9
	1630		*15.46*		*12.91*	#10
	1700	0.100				
	1730		*13.89*		*11.98*	#10
	1804			4.34		
	1830		*13.17*		*11.92*	#10
	1900	0.103				
	1930		*25.39*		*12.78*	#10
	2030		*26.42*		*30.11*	#11
	2100	0.120				
	2130		*29.49*		*28.42*	#11
	2230		*31.29*		*28.84*	#11
	2235			*10.99*		#11
	2300	0.114				
	2330				*30.29*	#11
13	0100	0.125				
	0130				*31.69*	#12
	0230		*38.69*		*27.80*	#12
	0300	0.135				
	0307			*13.22*		#12
	0330		*31.83*		*31.28*	#12
	0430		25.82		3.55	
	0500	[0.076]*				
	0530				0.32	
	0630				0.28	
	0700	0.130				
	0730				0.11	
	0737			0.08		
	0830		0.54		0.15	
	0900	0.112				
	0930		0.35		0.14	
	1030		0.29		0.14	
	1100	0.134				
	1130		2.04		0.12	
	1209			*3.02*		#13
	1230				*13.64*	#13
	1300	0.119				
	1330				*15.09*	#13
	1430		*15.47*		*15.63*	#13
	1500	0.129				
	1530		*12.05*		*5.62*	#14
	1630		*9.21*		*5.28*	#14
	1640			*2.00*		#14
	1700	0.136				
	1730		*7.58*		*5.29*	#14
	1830		*6.62*		*5.87*	#14
	1900	0.108				
	1930		*4.83*		*2.18*	#15
	2030		*3.50*		*1.91*	#15
	2100	0.103				
	2110			*0.72*		#15
	2130		*2.67*		*1.79*	#15
	2230		*2.77*		*1.92*	#15
	2300	0.124				
	2330				0.25	
14	0030				0.15	
	0100	0.201				
	0130				0.19	
	0141			0.10		
	0230				0.39	
	0300	1.057				
	0330				1.47	
	0430				2.24	
	0500	1.834				
	0530				2.52	
	0612			0.80		
	0630				2.29	
	0700	1.118				
	0730				1.47	
	0830				1.59	
	0900	1.185				
	1030				1.67	
	1043			0.64		
	1100	1.057				
	1130				1.87	
	1230				1.47	
	1300	0.934				
	1330		1.94		1.52	
	1430		1.89		1.64	
	1500	1.056				
	1515			0.59		
	1530		1.99		1.59	
	1630		1.90		1.62	
	1700	1.162				
	1730		1.91		1.56	
	1830		2.04		1.82	
	1900	1.232				
	1930		2.07		1.77	
	1949			0.70		
	2030		2.17		2.01	
	2100	1.247				
	2130		2.11		2.01	
	2230		2.14		2.11	
	2300	1.316				
	2330		2.08		2.02	
15	0023			0.68		
	0030		2.05		1.84	
	0100	1.239				
	0130		1.88		1.76	
	0230		1.90		1.68	
	0300	1.154				
	0330		1.83		1.63	
	0430		1.83		1.61	
	0456			0.64		
	0500	1.049				
	0530		1.75		1.65	
	0630		1.79		1.53	
	0700	1.030				
	0730		1.82		1.71	
	0830		1.86		1.76	
	0900	[0.906]				
	0930		1.75	0.59	1.53	
	1030		1.69		1.34	
	1100	0.722				
	1130		1.63		1.26	
	1230		1.55		1.24	
	1300	0.600				
	1330		1.33		0.96	
	1404			0.38		
	1430		1.18		0.98	
	1500	0.671				
	1530		1.13		1.03	
	1630		1.09		0.98	
	1700	0.561				
	1730		1.17			
	1830		1.01		0.84	
	1840			0.37		
	1900	0.591				
	1930		1.37		0.85	
	2030		1.22		0.94	
	2100	0.633				
	2130		1.02		1.03	
	2230		1.09		1.10	
	2300	0.649				
	2316			0.38		
	2330		0.99		0.95	
16	0030		1.05		0.92	
	0100	0.505				
	0130		1.07		0.81	
	0230		1.07		0.87	
	0300	0.518				
	0330		1.15		0.98	
	0352			0.36		
	0430		1.22		1.08	
	0500	0.578				
	0530		1.32		1.21	
	0630		1.42		1.26	
	0700	[0.568]				
	0730		1.44		1.41	
	0827			0.46		
	0830		1.45		1.08	
	0900	0.567				
	0930		1.29		1.05	
	1030		1.22		0.80	
	1100	0.526				
	1130		1.06		0.77	
	1230		1.08		0.63	
	1300	0.435				
	1302			0.30		
	1330		0.95		0.81	
	1430		0.97		0.78	
	1500	0.481				
	1530		0.92		0.79	
	1630		1.00		0.92	
	1700	0.455				
	1730		1.01		0.93	
	1740			0.97		
	1830		1.02		0.80	
	1900	0.439				
	1930		0.99		0.80	
	2030		0.98		0.71	
	2100	0.393				
	2130		0.84		0.73	
	2218			0.25		
	2230		0.80		0.61	
	2300	0.338				
	2330		0.72		0.48	
17	0030		0.66		0.48	
	0100	0.354				
	0130		0.69			
	0230		0.67		0.54	
	0255			0.22		
	0300	0.307				
	0330		0.57		0.46	
	0430		0.48		0.15	
	0500	0.182				
	0530		0.44		0.37	
	0630		0.48		0.42	
	0700	0.156				
	0730		0.40		0.14	
	0731			0.12		
	0830		0.44		0.30	
	0900	0.236				
	0930		0.46		0.43	
	1030		0.50		0.43	
	1100	0.253				
	1130				0.30	
	1230				0.36	
	1300	0.195				
	1330				0.33	
	1430				0.39	
	1500	0.182				
	1530				0.19	
	1630				0.22	
	1700	0.221				
	1730				0.24	
	1830				0.27	
	1900	0.222				
	1930				0.27	
	2030				0.26	
	2130				0.26	
	2230				0.32	
	2330				0.43	

a Approximate time for midpoint of the measurement interval at Greenwich Mean (Universal) Time (GMT).

b Mean ^222^Rn concentration for a measurement interval of 2 h. Associated uncertainties for concentrations in the range of about 0.07 Bq · m^−3^ to 0.2 Bq · m^−3^ were estimated and reported to be approximately ± 20 % (refer to text).

c Mean ^222^Rn concentration for a measurement interval of 1 h. Associated uncertainties (refer to text) are given in Fig. 6.

d Mean ^222^Rn concentration for a measurement interval of 2 h. Associated uncertainties were reported to range from 3.4 % to 3.9 %.

e Mean ^222^Rn concentration for a measurement interval of 1 h. Associated uncertainties (refer to text) are given in Fig. 5.

f Values in brackets denote those measurements having abnormally high ^212^Pb daughter concentrations which may suspect local land influences.

g Values with asterisks denote those suspected to be influenced by rainfall.

h Values in italics are those obtained during the indicated standard addition number.
